# Supplementary material for: Egg-sac-brooding wolf spiders show flexible hatchling emergence and context-dependent escape performance
Source: Biol Open. 2025 Nov 7;14(11):bio062232. doi: 10.1242/bio.062232 (PMC12641480; doi:10.1242/bio.062232)
Supplement: Supplementary information [file biolopen-14-062232-s1.pdf]

File S1.

Available for download at
https://journals.biologists.com/bio/article-lookup/doi/10.1242/bio.062232#supplementary-data
